# Supplementary material for: Association of ABCG2 421G>T (rs2231142) Polymorphism with rosuvastatin induced adverse effects in dyslipidemic patients: Implication for personalized medicine
Source: PLoS One. 2025 Oct 17;20(10):e0334600. doi: 10.1371/journal.pone.0334600 (PMC12533910; doi:10.1371/journal.pone.0334600)
Supplement: S1 — (DOCX) [file pone.0334600.s001.docx]

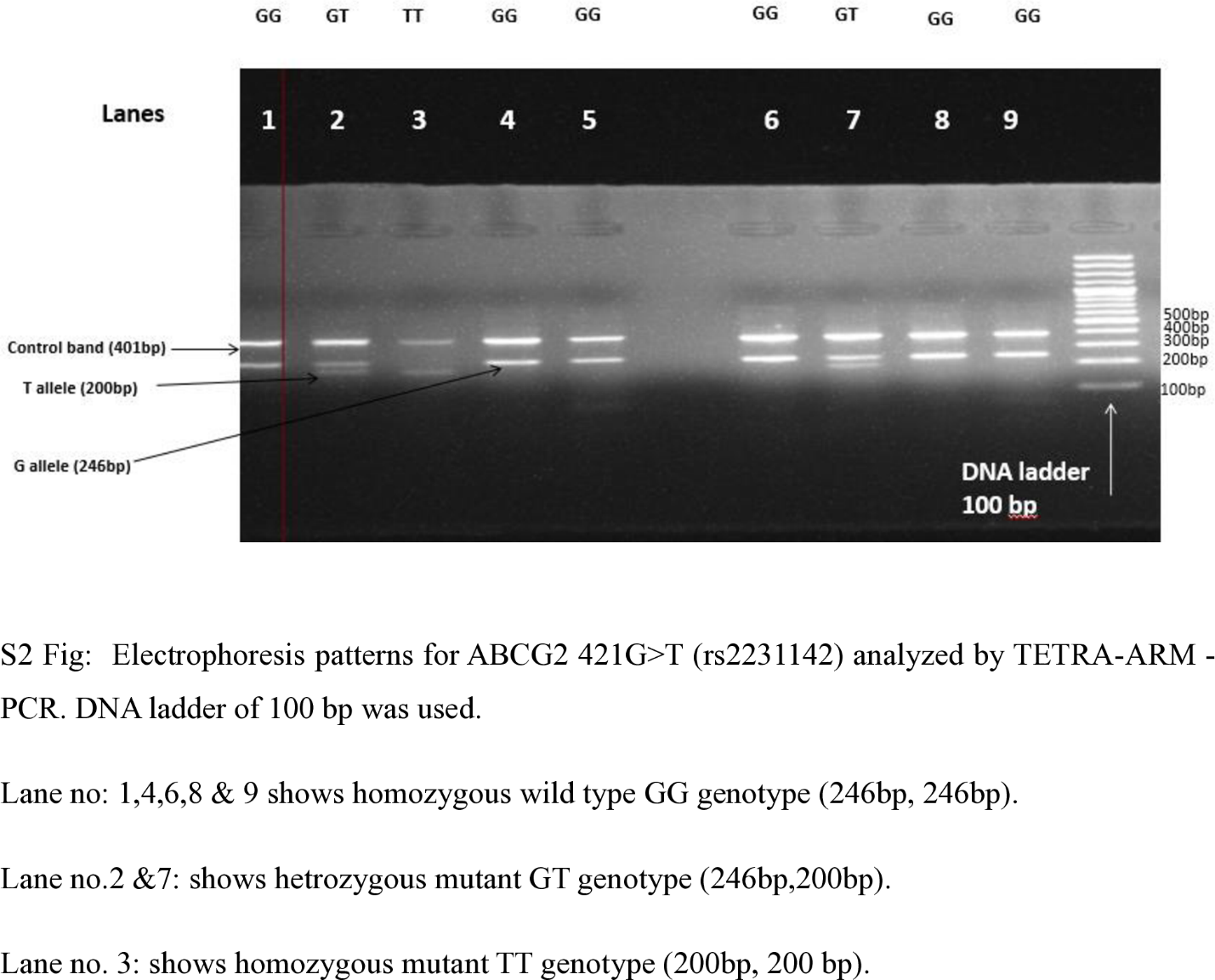


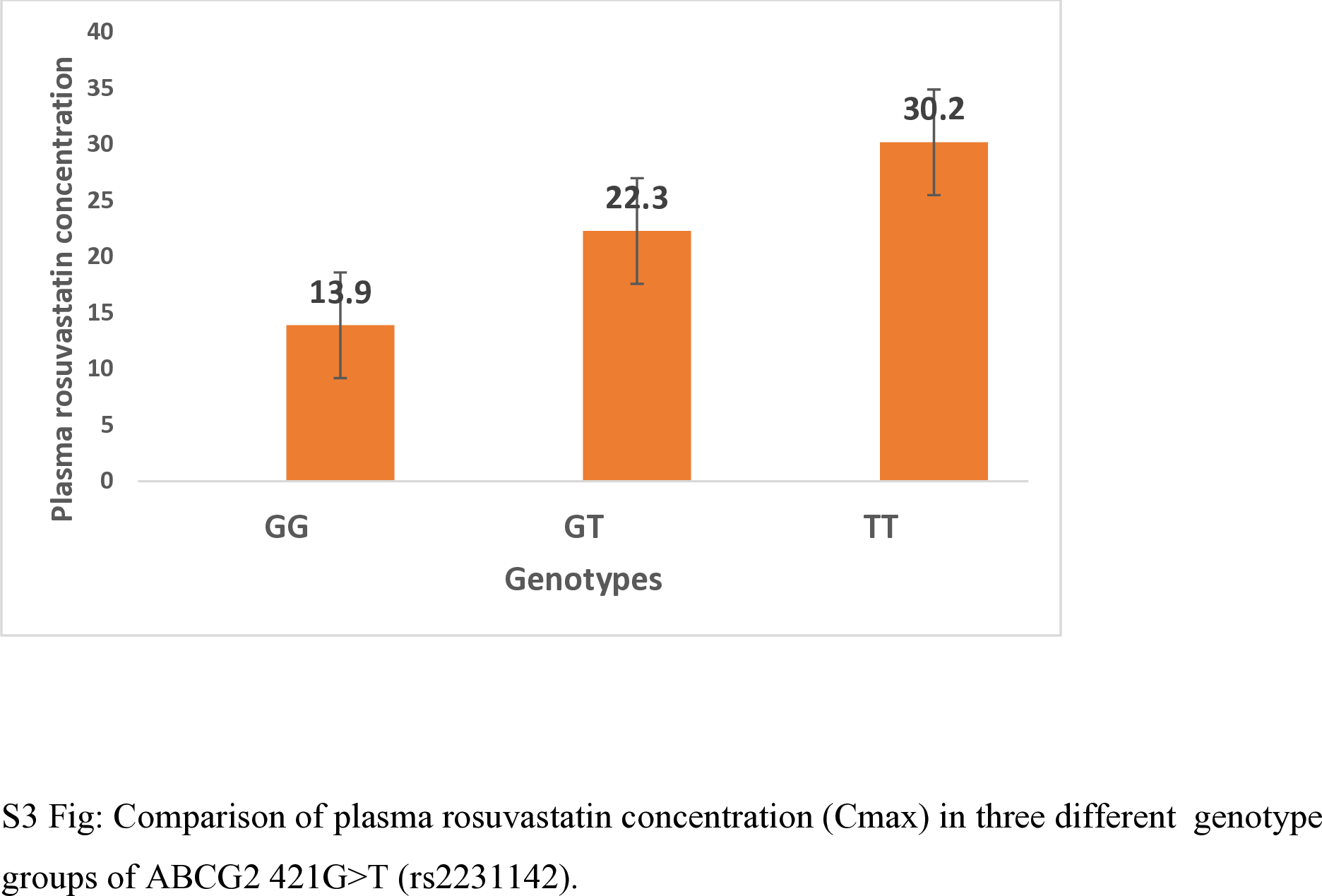


S1 Table: Baseline characteristics of study population.

| Characteristics | | Mean±SD | n= 376 | %age |
| --- | --- | --- | --- | --- |
| Gender | Male |  | 215 | 57.2 |
|  | Female |  | 161 | 42.8 |
| Age (years)  (30-70 years) | | 54.47±9.58 | 376 |  |
| Ethnicities | Punjabi | | 182 | 48.4 |
|  | Sindhi | | 42 | 11.2 |
|  | Pathan | | 61 | 16.2 |
|  | Balochi | | 34 | 9.0 |
|  | Kashmiri | | 35 | 9.3 |
|  | Gilgiti | | 22 | 5.9 |
| Weight (kg) | | 81.1±9.57 |  |  |
| Height (cm) | | 167.63±7.72 |  |  |
| BMI (kg/m2) | | 29.04±4.2 |  |  |
| Waist circumference (cm) | | 97.23±5.51 |  |  |

S2 Table: Genotype and allele frequencies of ABCG2 421 G>T

| Observed alleles | | | Observed Genotypes | | | Expected Genotypes | HWE- *p* value |
| --- | --- | --- | --- | --- | --- | --- | --- |
| Alleles | No of Alleles (n=748) | Observed frequency of Alleles (%) | Genotypes | Total no of patients (n=374) | Frequency  (% age) |  | 0.07^#^ |
| G | 546 | 0.73  (73%) | GG | 205 | 54.5 | 198 |  |
| T | 206 | 0.27  (27%) | GT | 136 | 36.2 | 150 |  |
|  | | | TT | 35 | 9.3 | 28 |  |

^#^ = HWE –*p* value >0.05 means that observed frequencies were in accordance with

expected values.

S3 Table : Comparison of biochemical data between pretreatment and post treatment visits.

| Parameters | Baseline values  (Mean±SD) | Post-treatment values(Mean±SD) | % age change | *p*-value |
| --- | --- | --- | --- | --- |
|  |  |  |  |  |
| CPK(U/L) | 100.16±26.28 | 272.42±477.8 | 156.75±440.09 | <0.001* |
| ALT (U/L) | 23.38±7.4 | 33.91±29.9 | 46.34±105.28 | <0.001* |
| ALP | 79.10±18.27 | 104.41±65.2 | 39.0±90.5 | <0.001* |
| S. urea (mg/dL) | 25.13±6.34 | 29.22±11.84 | 19.24±48.2 | <0.001* |
| S. creatinine (mg/dL) | 0.87±0.34 | 1.0±0.48 | 22.41±63.5 | <0.001* |
| S. sodium (mmol/L) | 138.39±2.4 | 138.39±2.9 | 0.264±2.79 | 0.108# |
| S. Potassium  (mmol/L) | 4.17±0.426 | 4.17±0.52 | 0.73±14.70 | 0.95# |

Data were expressed as mean±SD.

Statistical analysis was performed by paired t test.

*=  *p*<0.05

#= *p* >0.05

S4 Table: ABCG2 G>T (rs2231142) genetic models showing association with rosuvastatin induced adverse effects

| Model | Genotype | Rosuvastatin induced adverse effects absent | Rosuvastatin induced adverse effects present | OR  95% CI | *p-*value |
| --- | --- | --- | --- | --- | --- |
| Co-dominant | G/G | 183 (68%) | 22(20.6%) | 1.00 (Reference) | |
|  | G/T | 83 (30.9%) | 53 (49.5%) | 5.45  (3.09-9.62). | <0.0001 |
|  | T/T | 3(1.1%) | 32(29.9%) | 88.51  (24.84-315.44) |  |
| Dominant | G/G | 183(89.4%) | 22(41.2%) | 1.00 (Reference) | |
|  | G/T+T/T | 86(10.6%) | 85(58.8%) | 8.45  (4.91-14.52) | <0.0001 |
| Recessive | G/G+G/T | 266(98.9%) | 75(70.1%) | 1.00 (Reference) | |
|  | T/T | 3(1.1%) | 32 (29.9%) | 37.29  (11.06-125.78) | <0.0001 |
| Over-dominant | G/G+T/T | 186(69.1%) | 54(50.5%) | 1.00 (Reference) | |
|  | G/T | 83(30.9%) | 53(49.5%) | 2.26  (1.42-3.6) | <0.001 |
