# Supplementary figures and images for: Association of ABCG2 421G>T (rs2231142) Polymorphism with rosuvastatin induced adverse effects in dyslipidemic patients: Implication for personalized medicine
Source: PLoS One. 2025 Oct 17;20(10):e0334600. doi: 10.1371/journal.pone.0334600 (PMC12533910; doi:10.1371/journal.pone.0334600)

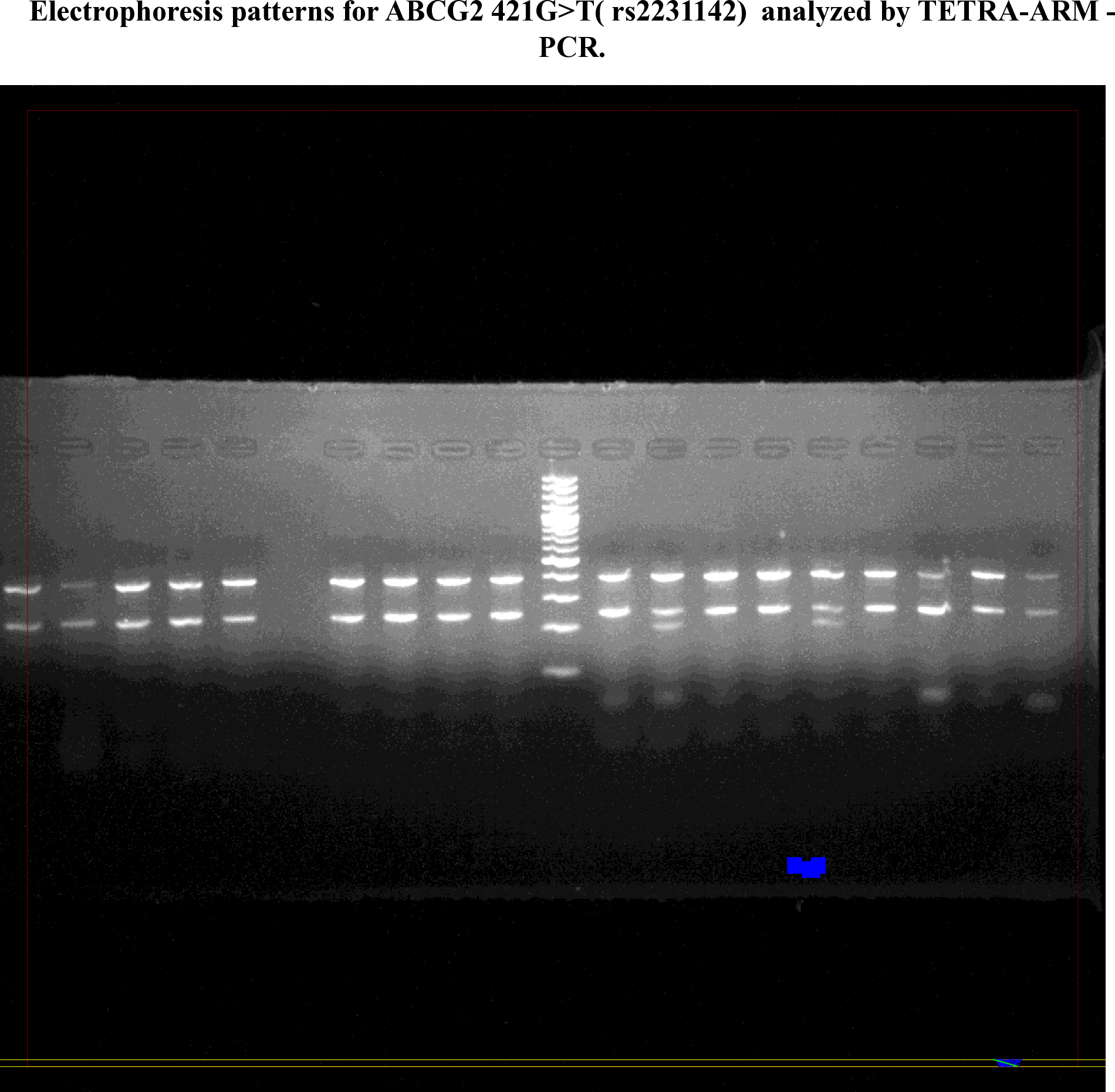


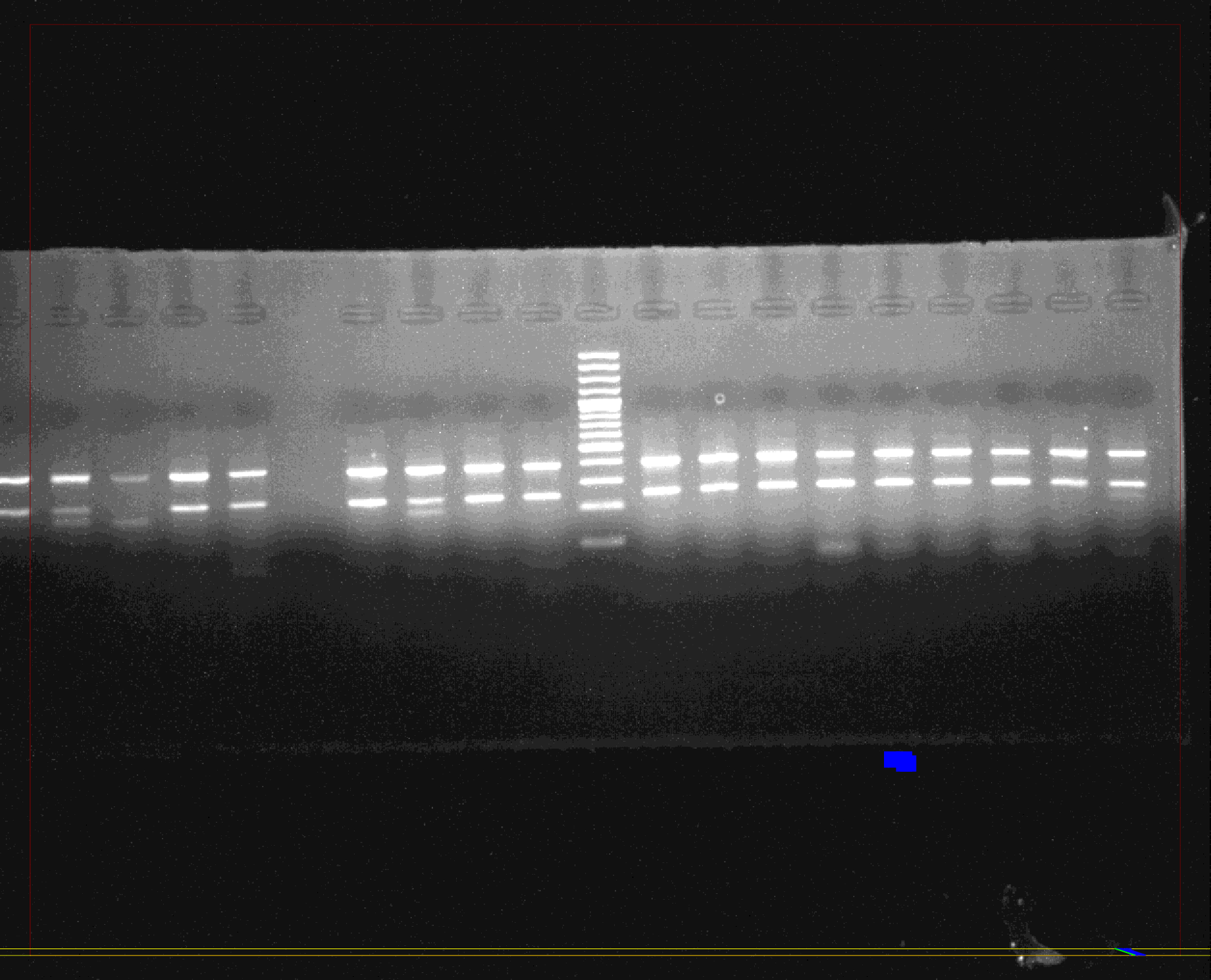


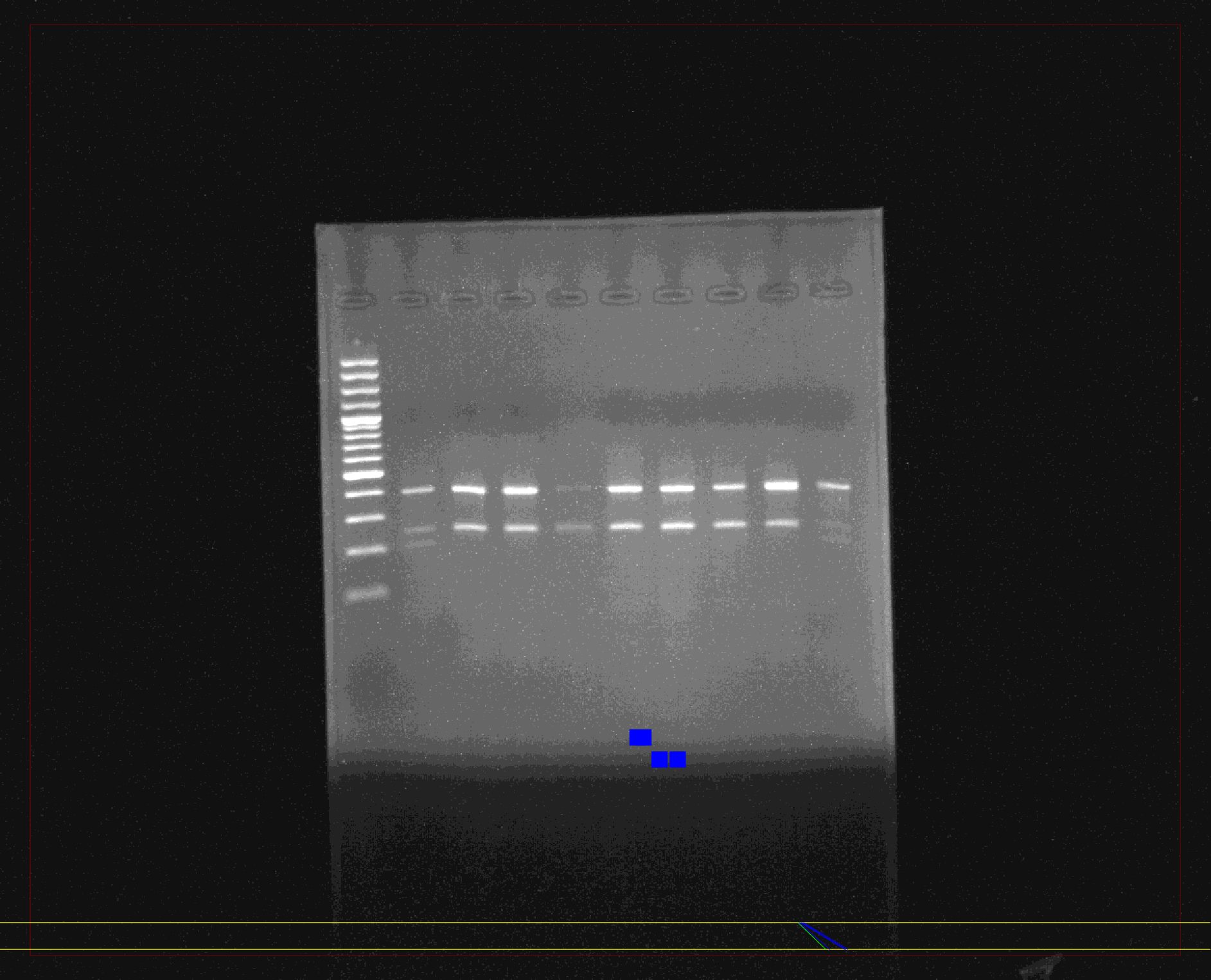


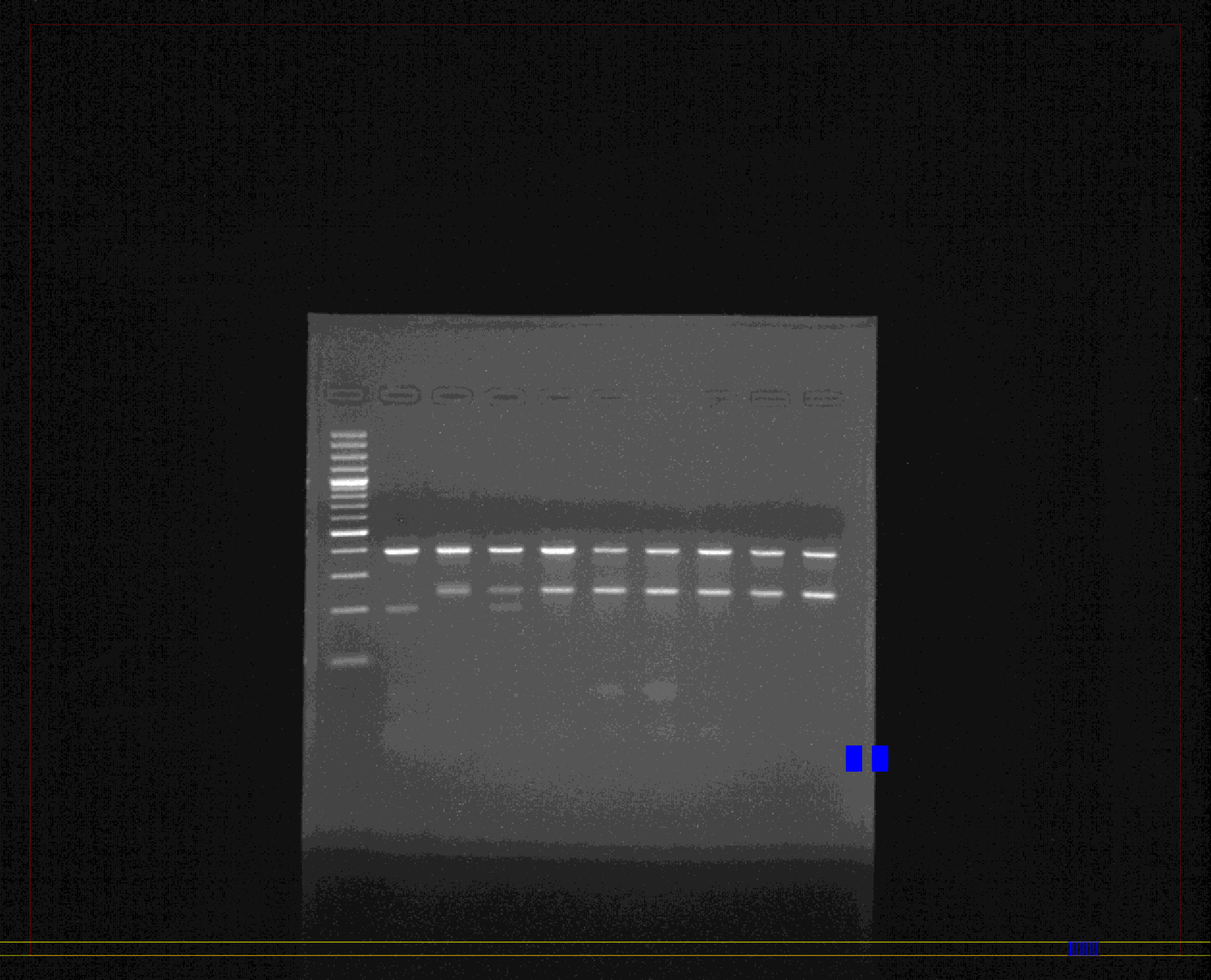


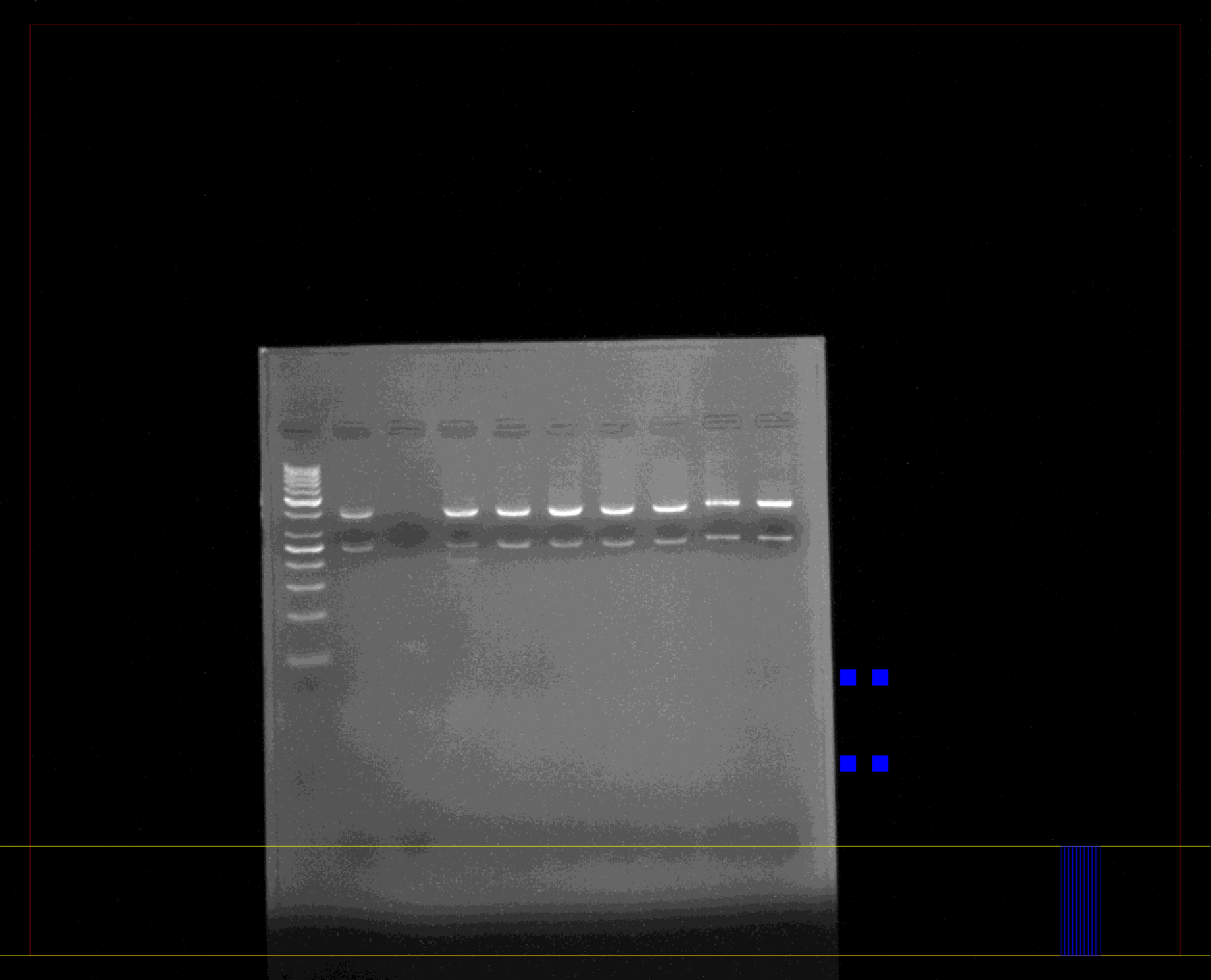

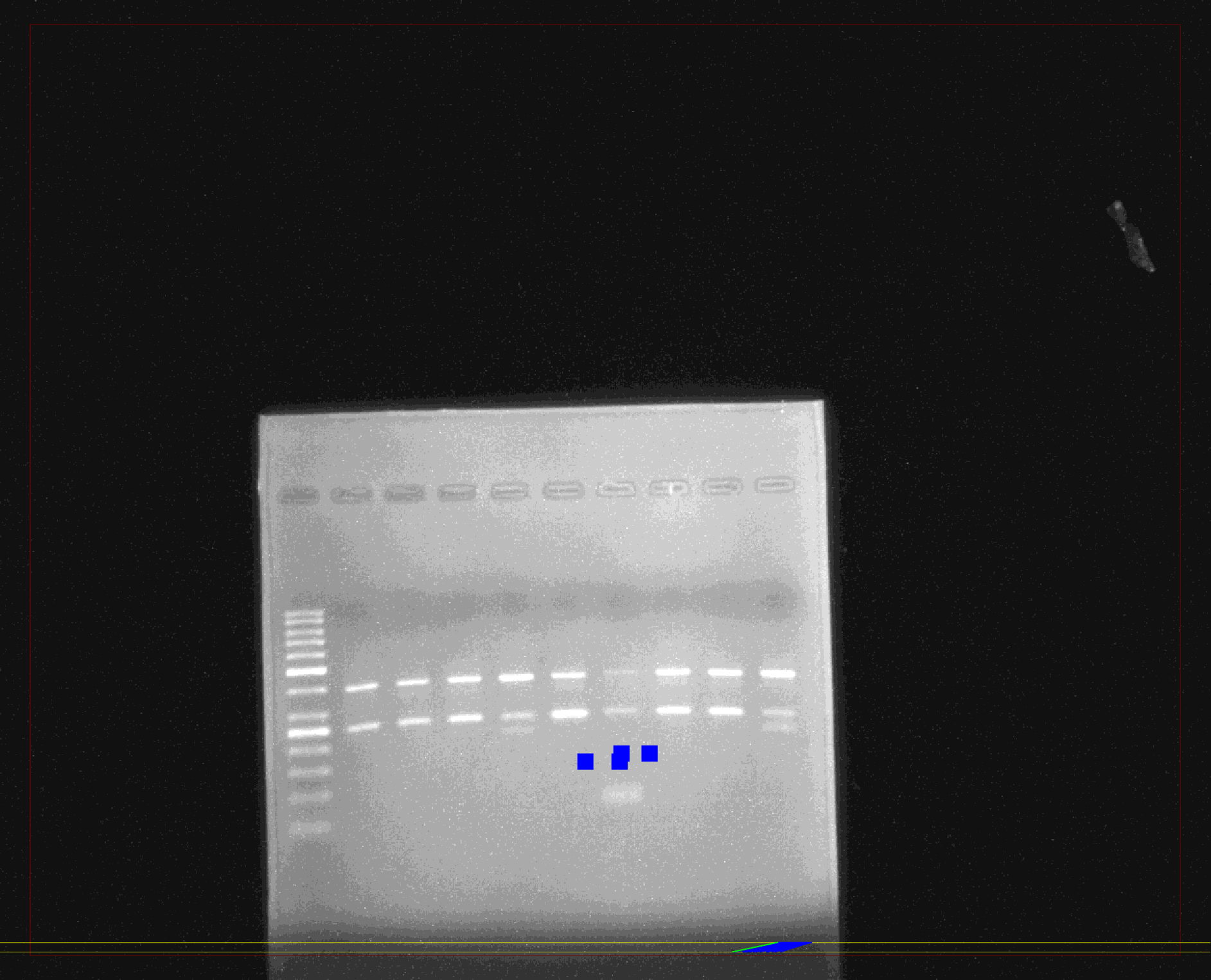

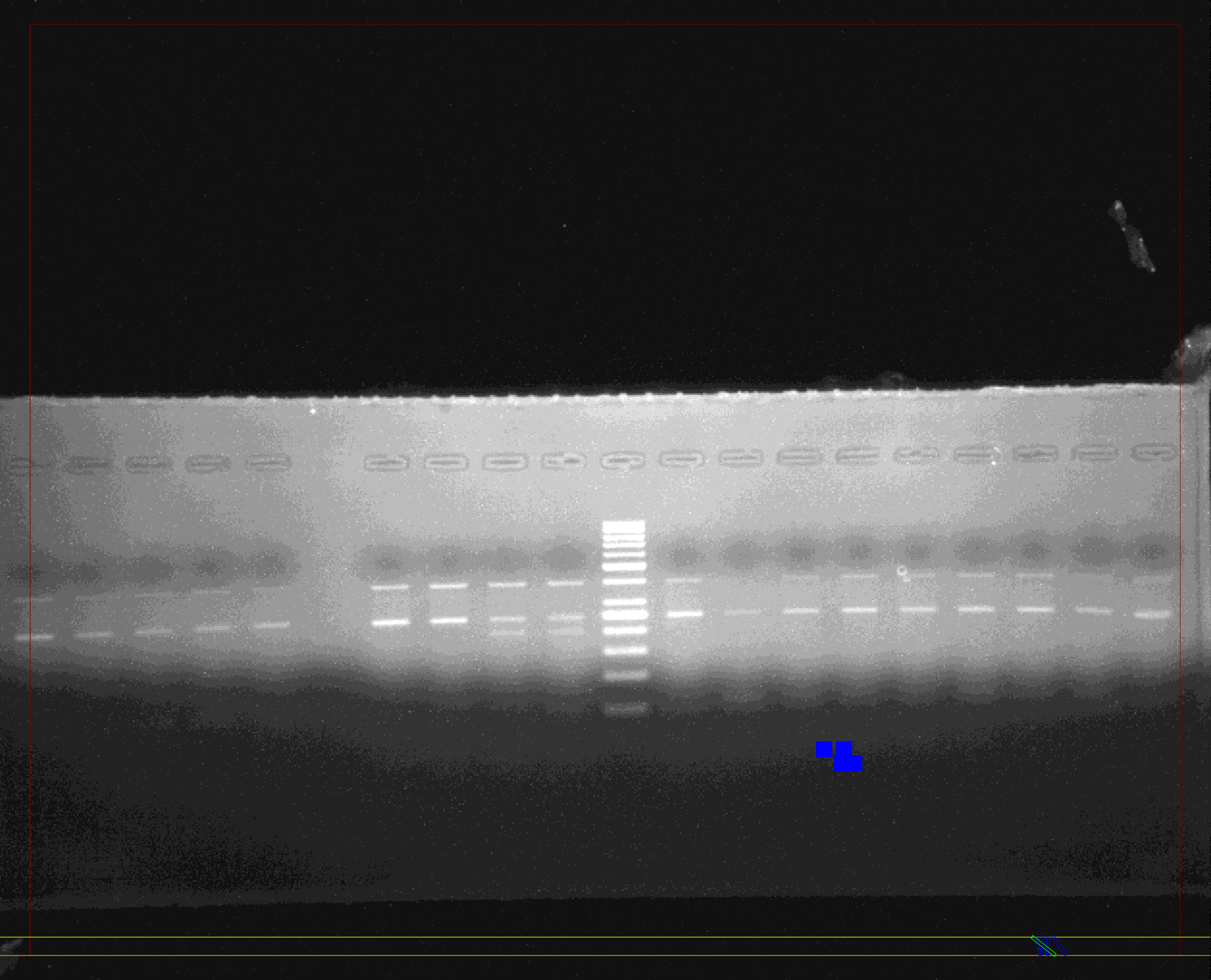

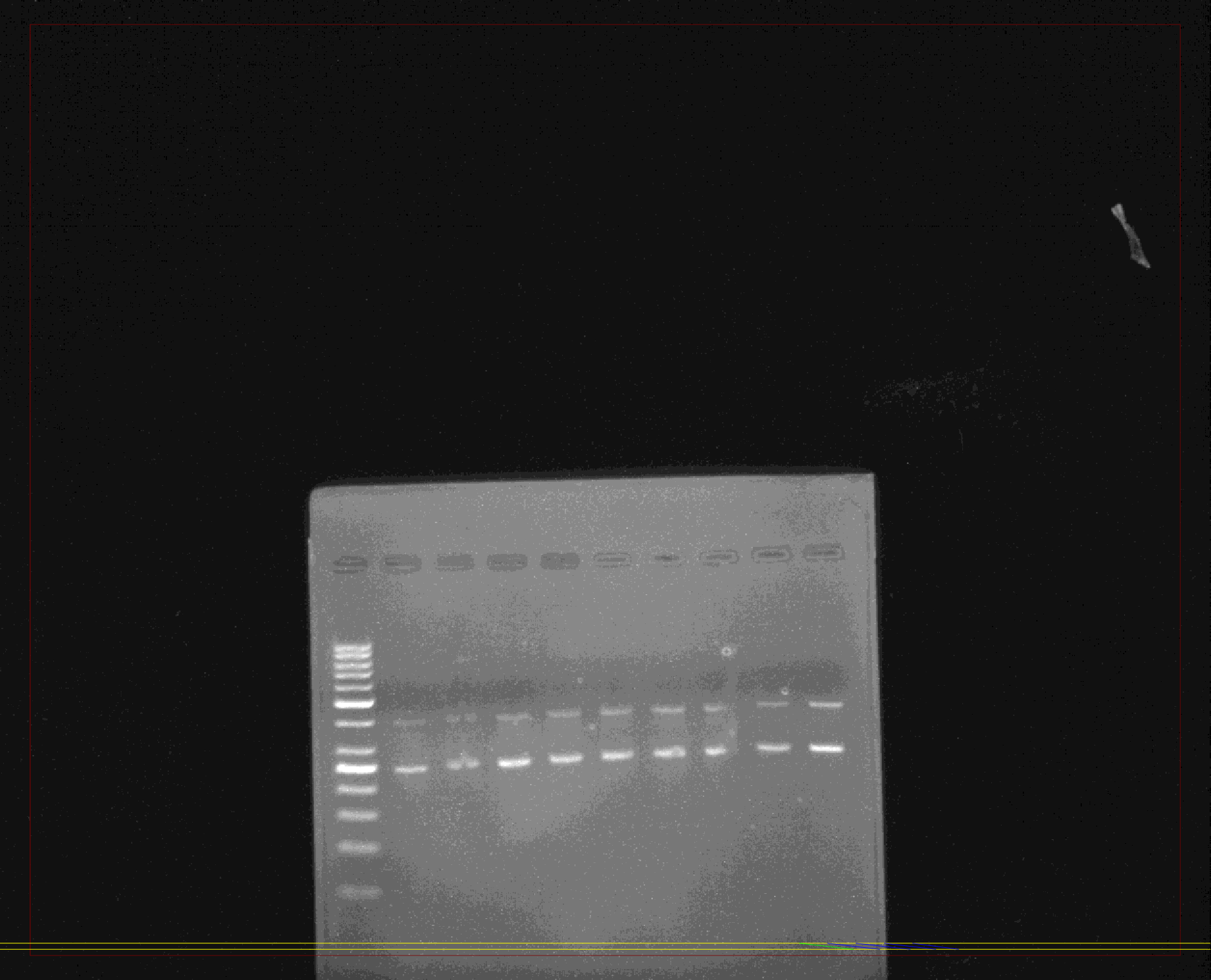

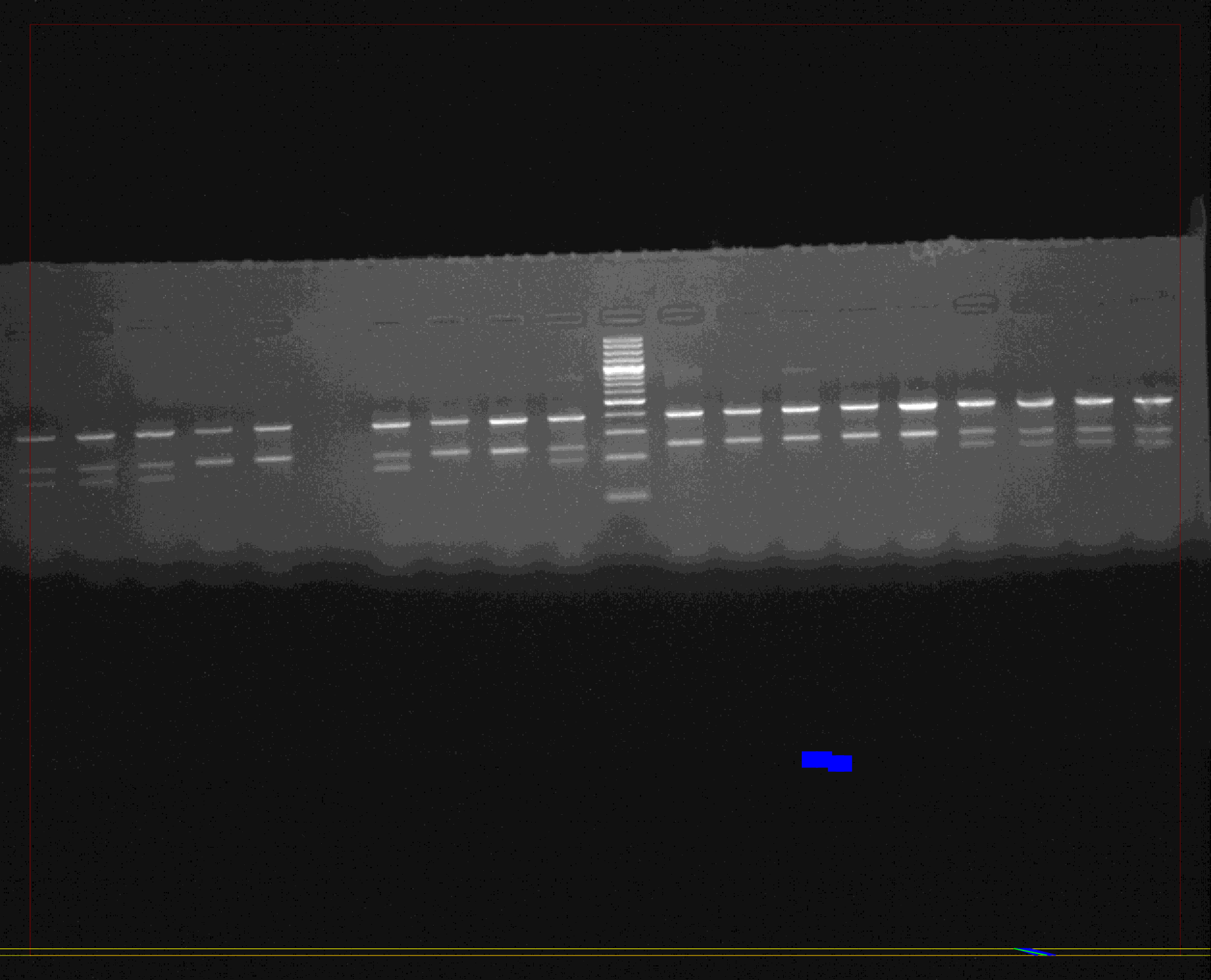

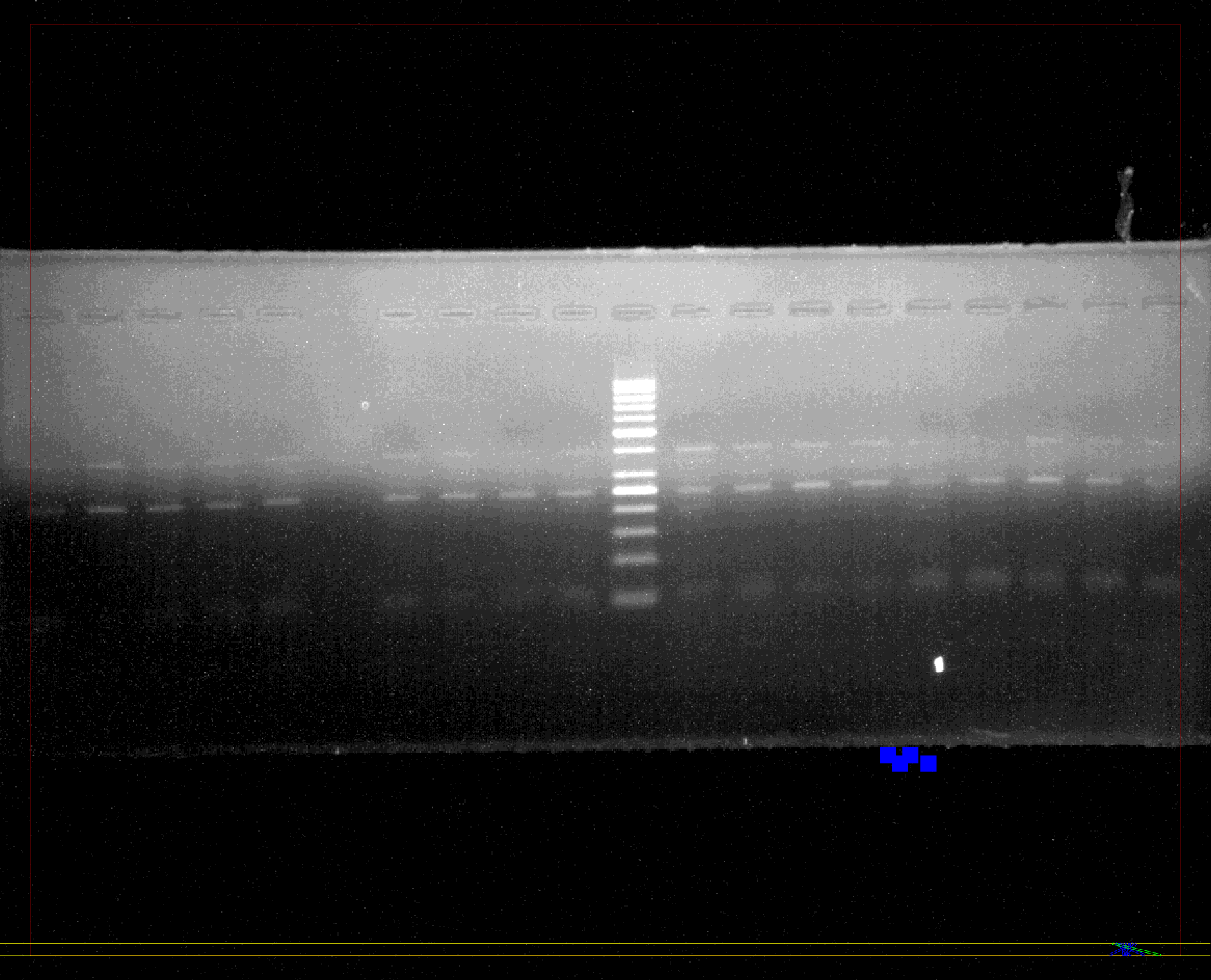

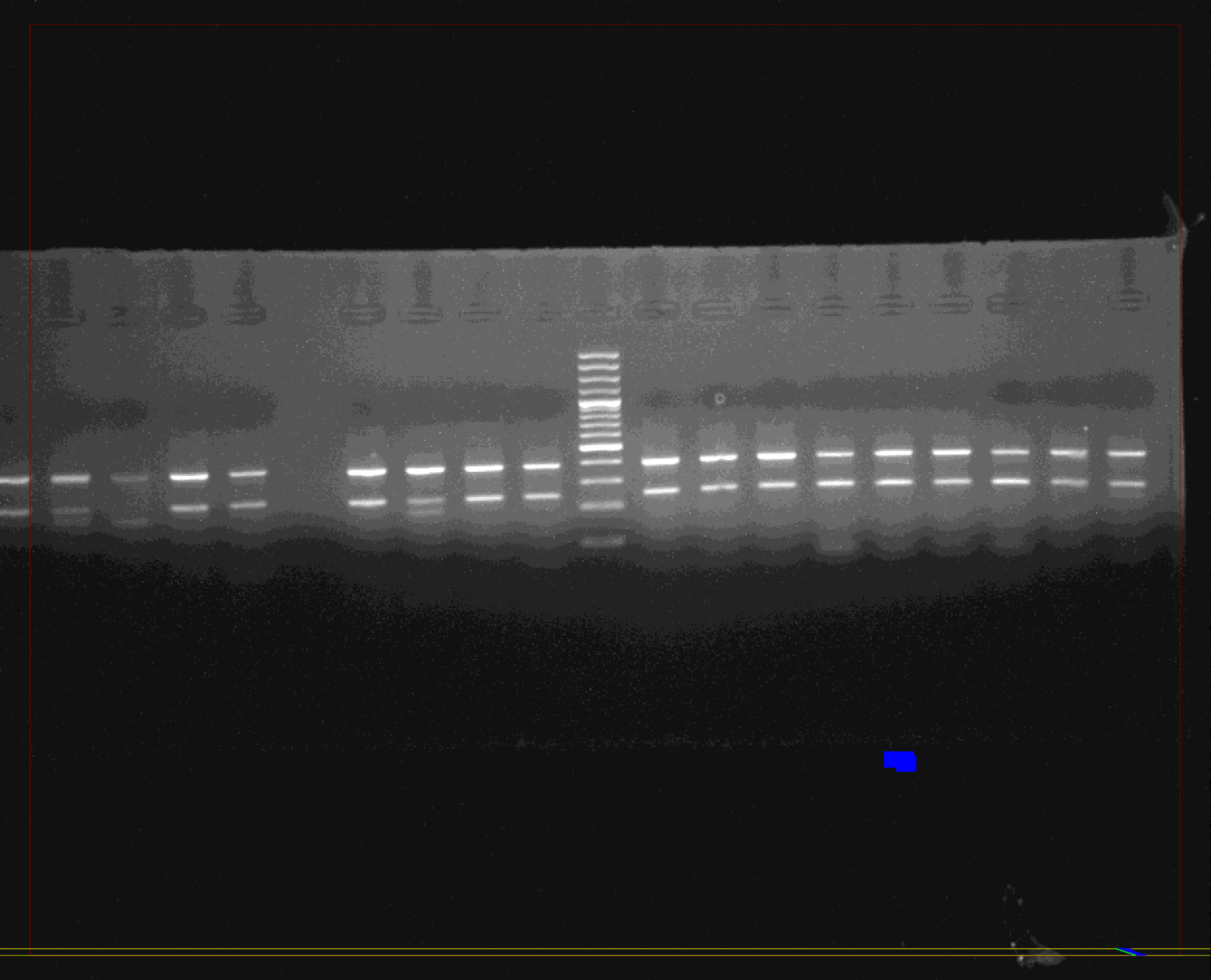

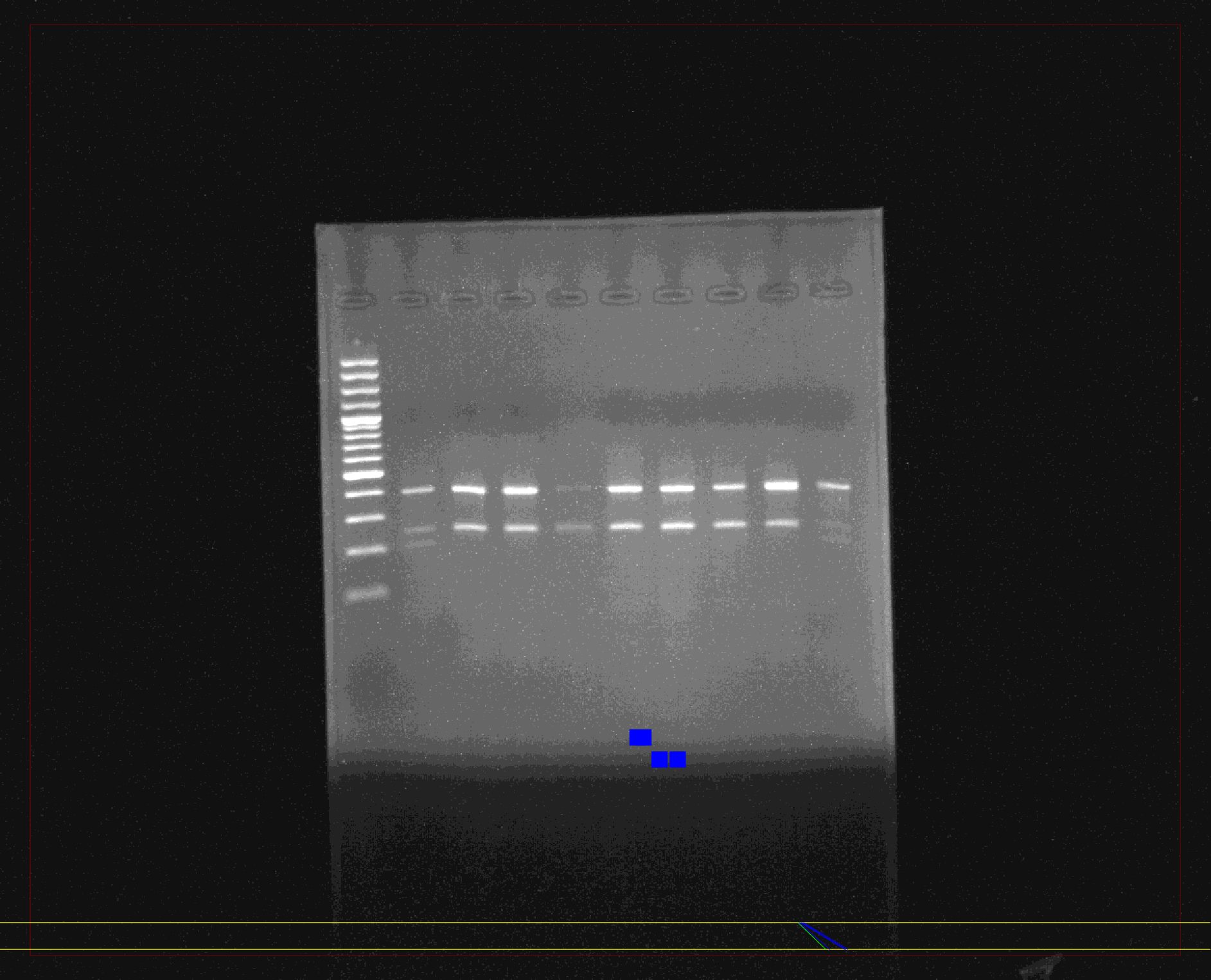

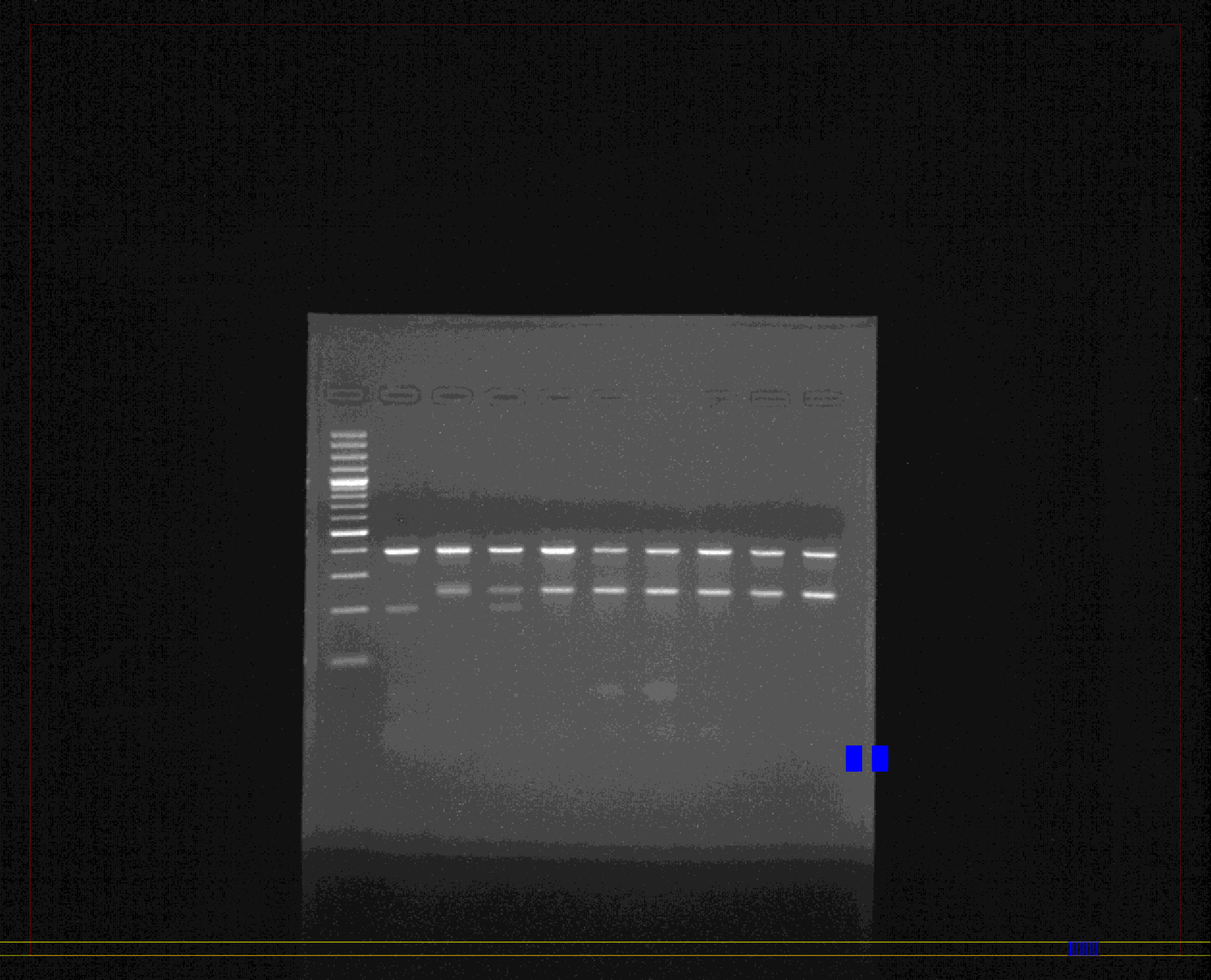

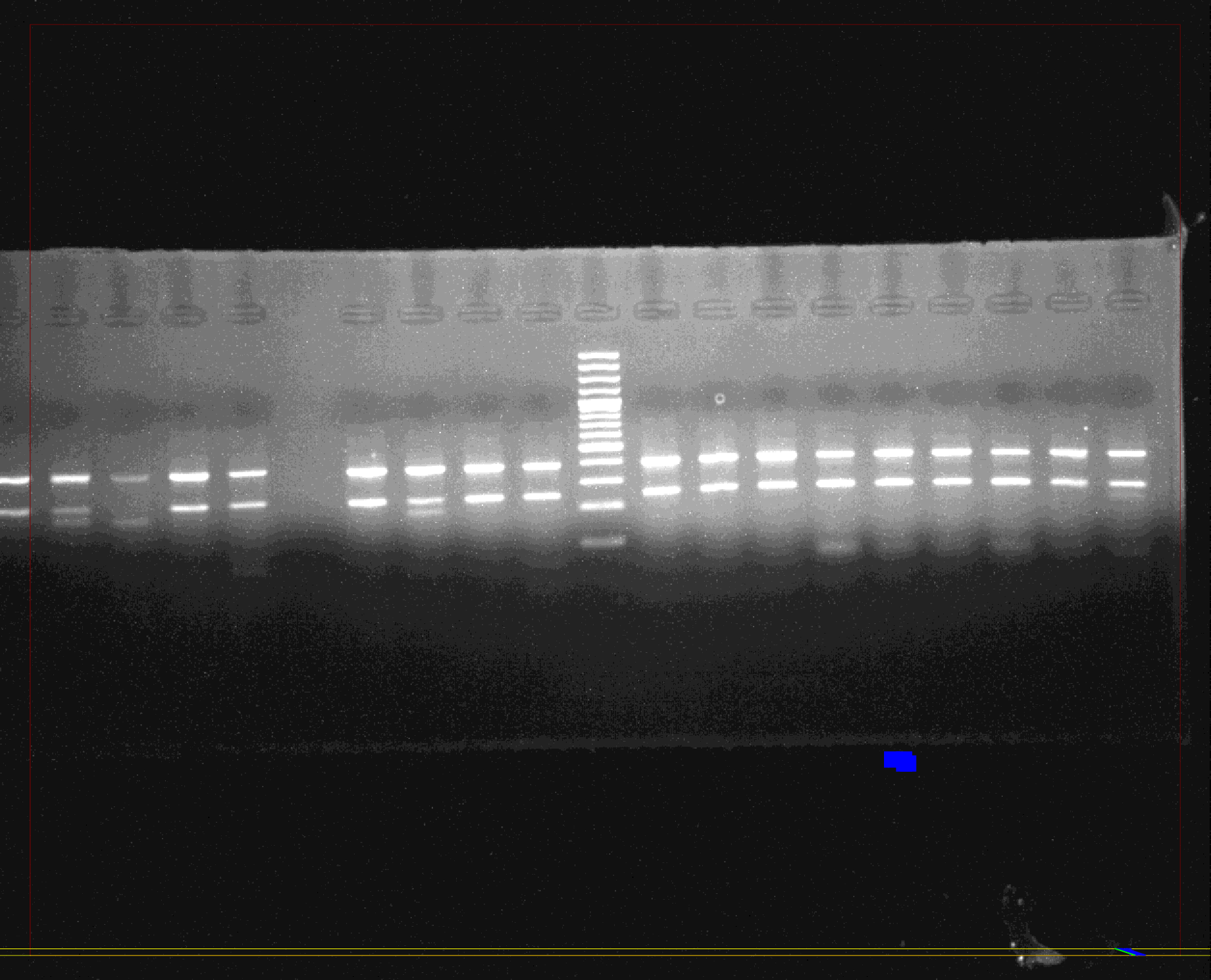

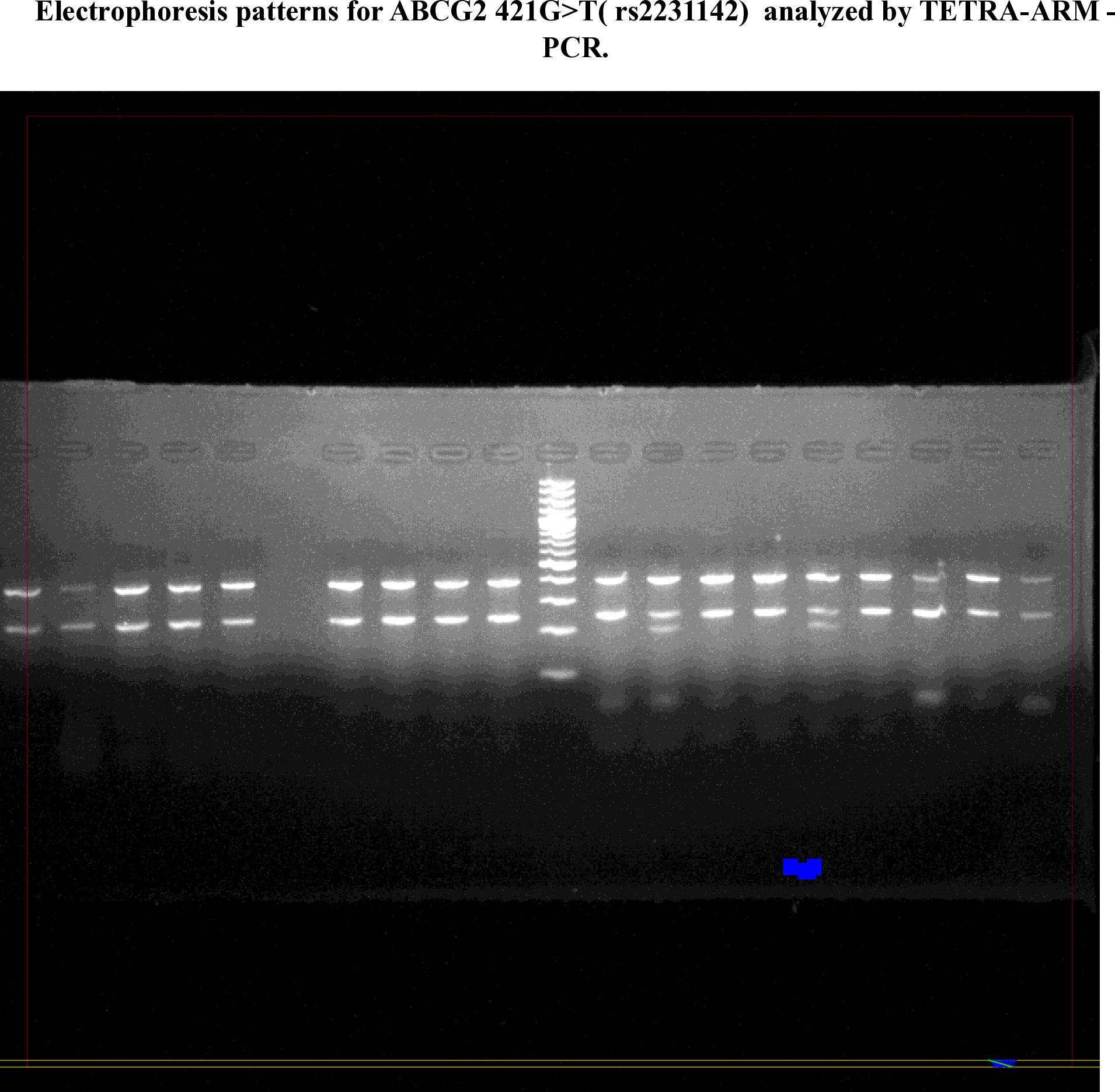

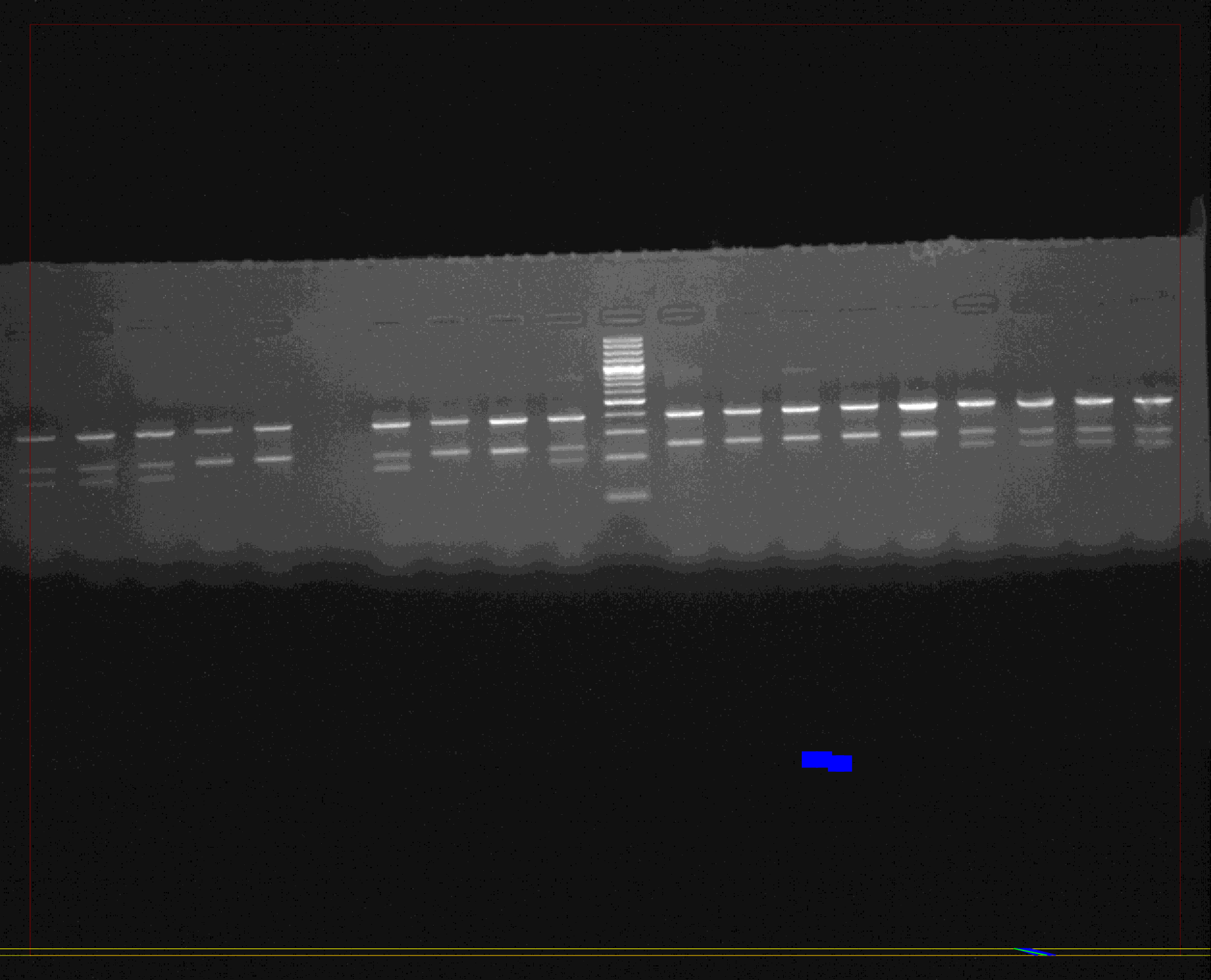

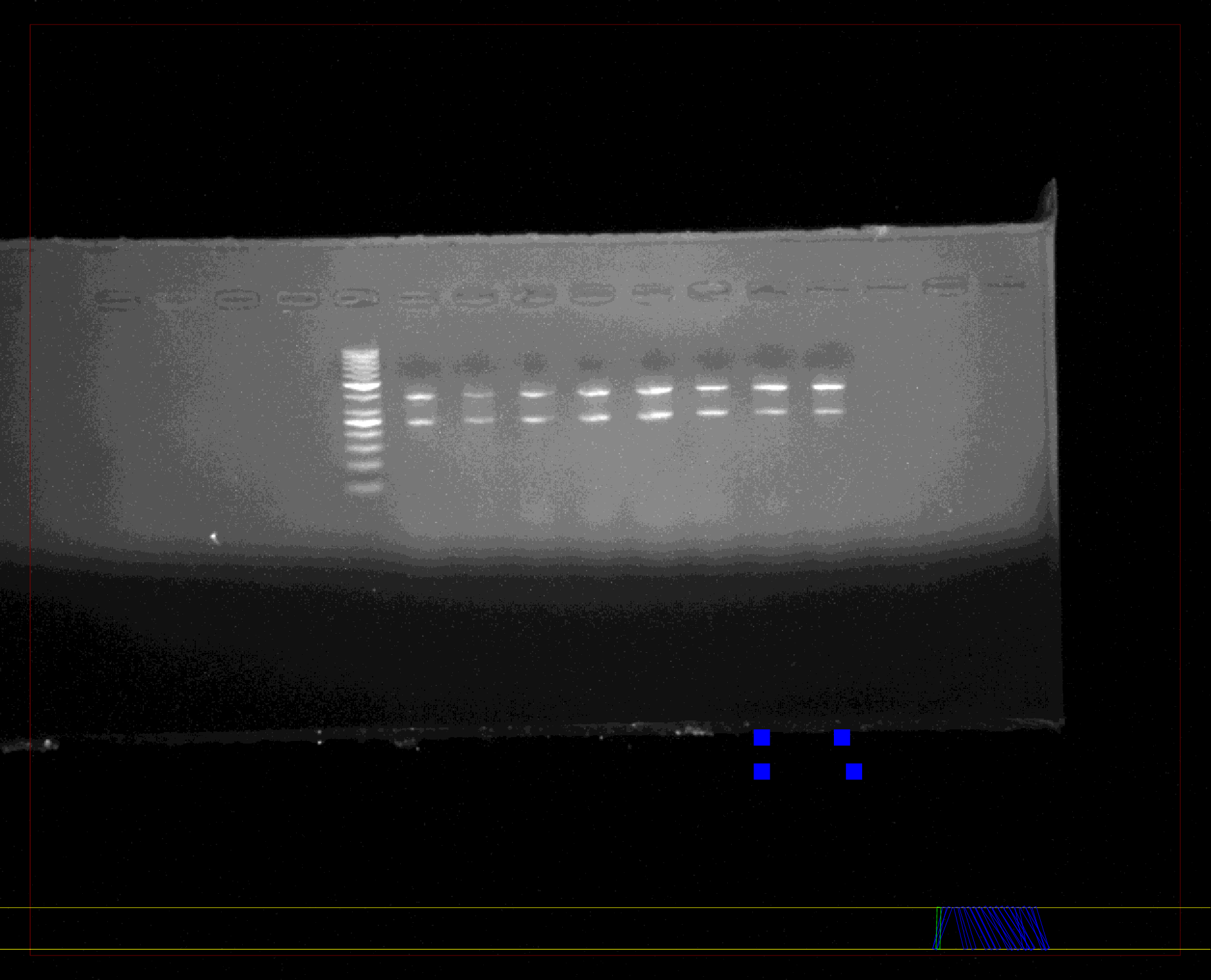

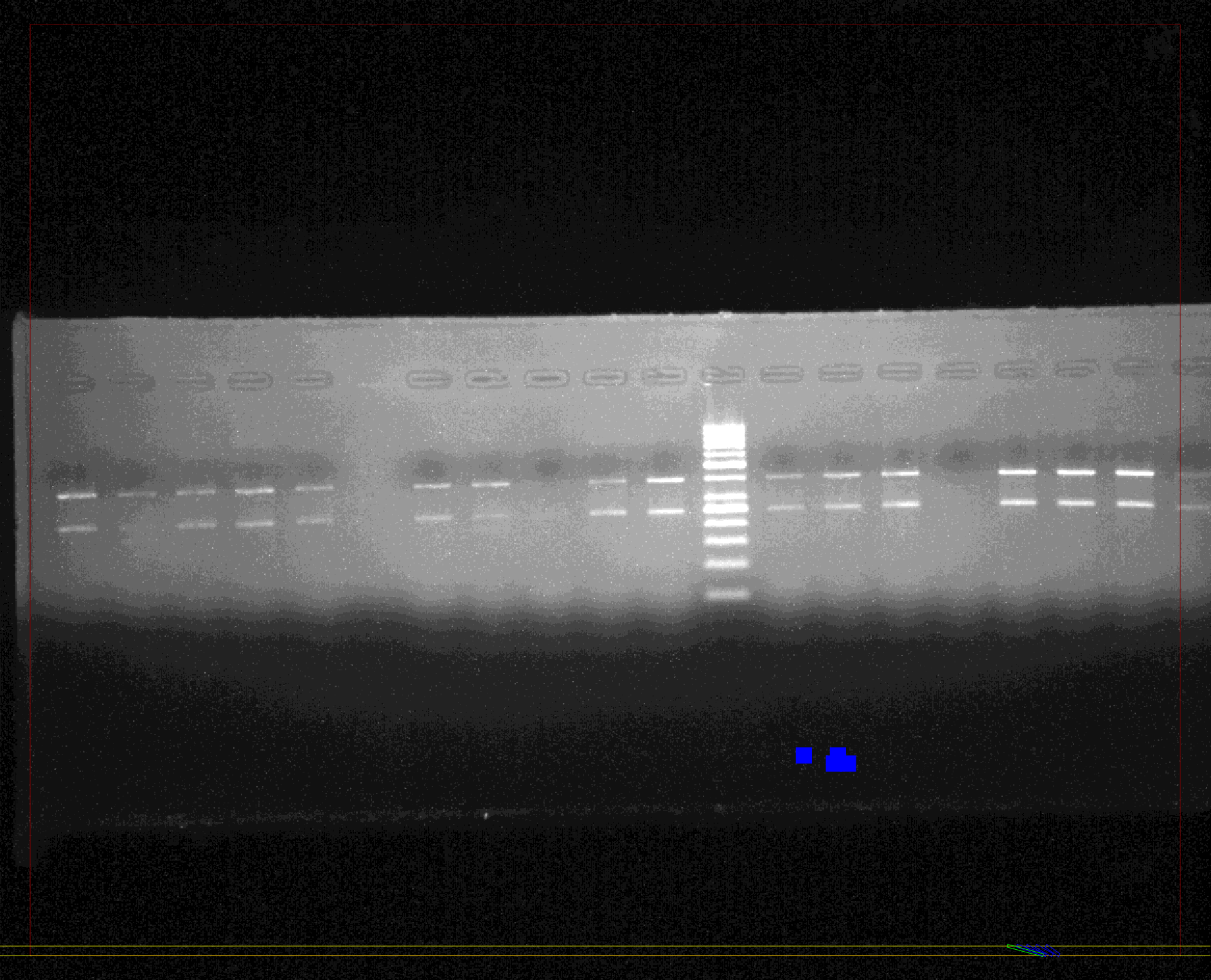

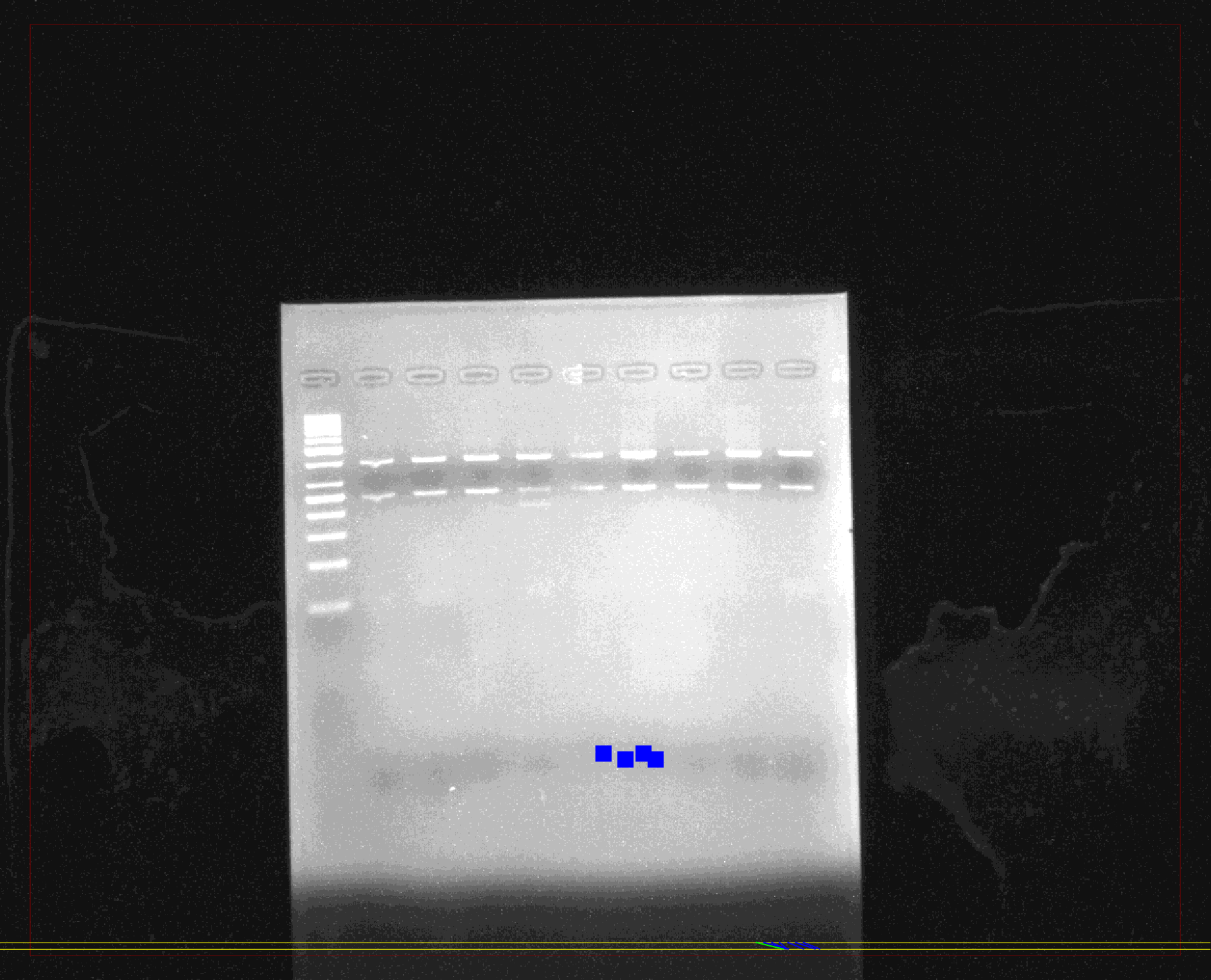

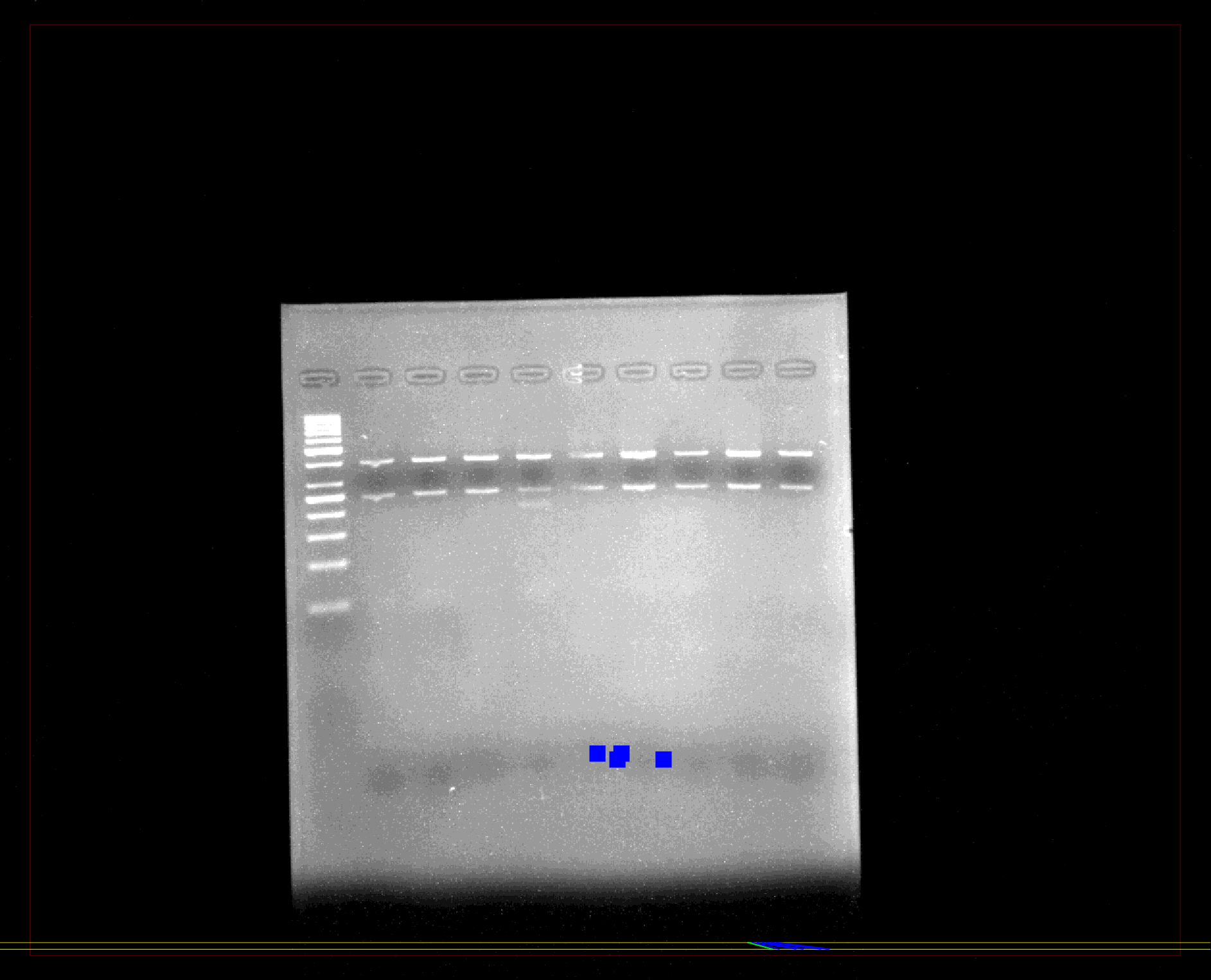

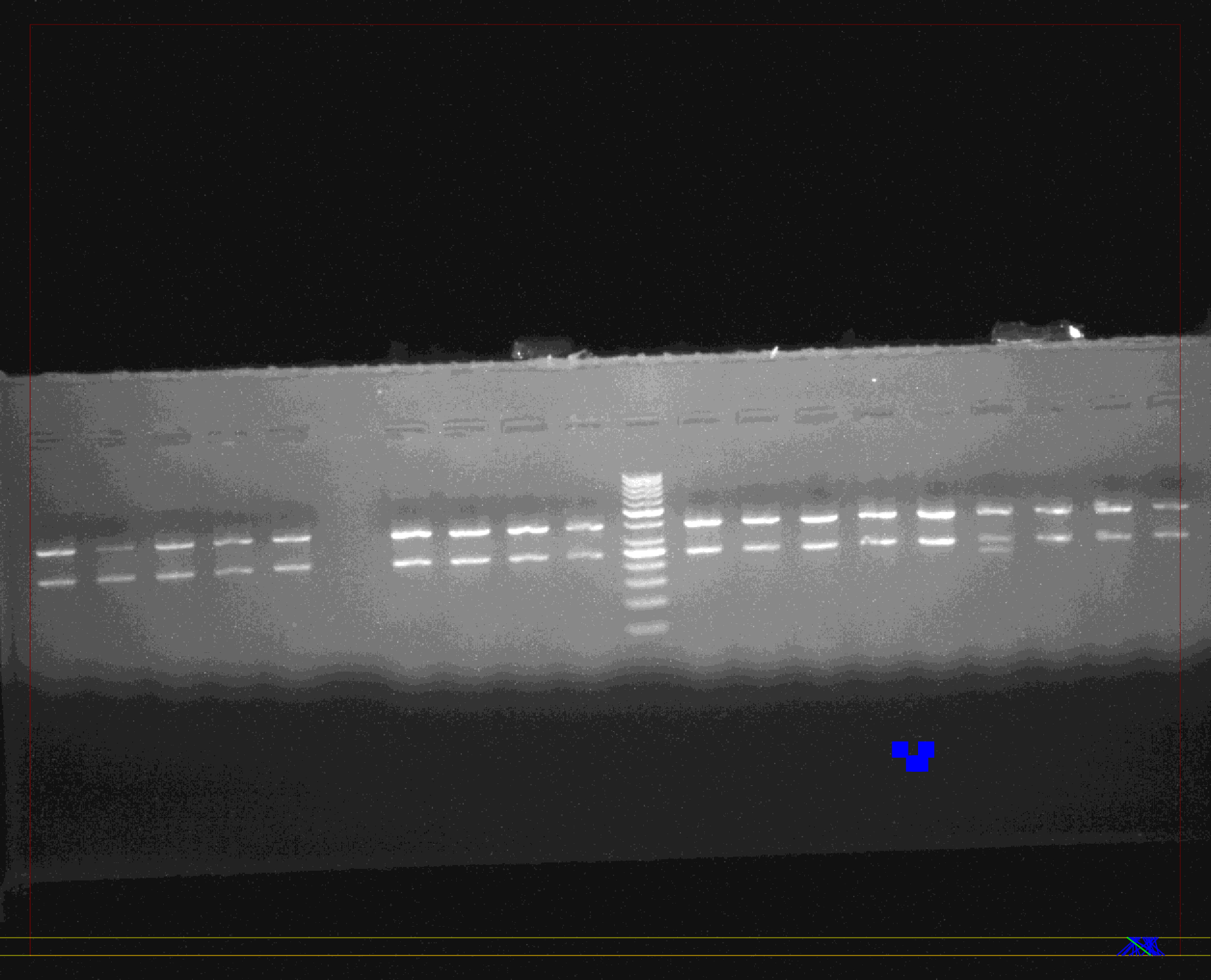

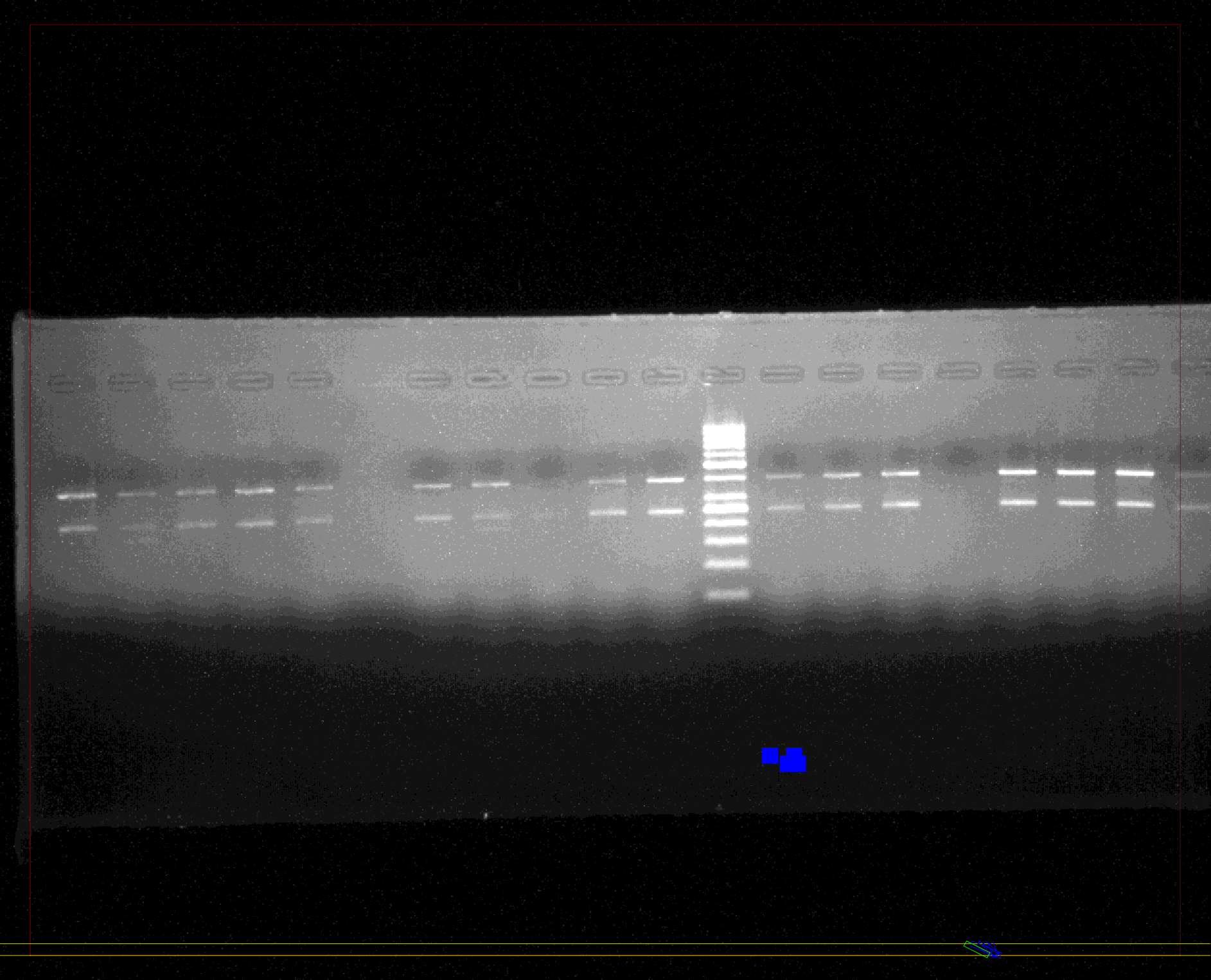

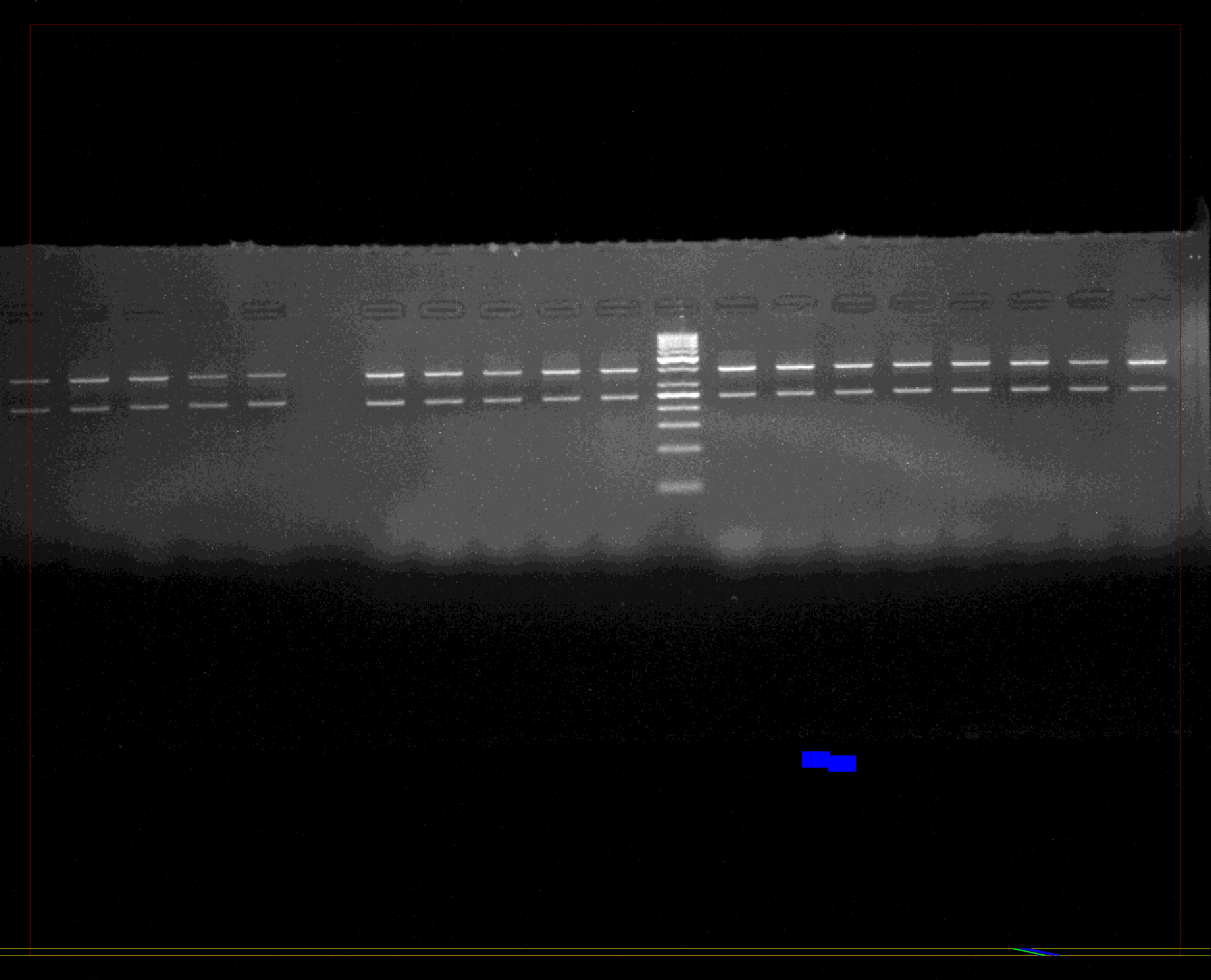

Supplement: S2 — (DOCX) [file pone.0334600.s002.docx]
